# System-Level Quality Improvement Initiatives for Tobacco Use in a Safety-Net Health System During the COVID-19 Pandemic

**DOI:** 10.1177/21501319221107984

**Published:** 2022-06-24

**Authors:** Kara Chung, Henry Rafferty, Leslie W. Suen, Maya Vijayaraghavan

**Affiliations:** 1 University of California, San Francisco, San Francisco, CA, USA; 2 San Francisco Department of Public Health, San Francisco, CA, USA; 3 San Francisco Veteran Affairs Medical Center, San Francisco, CA, USA

**Keywords:** quality improvement, tobacco use interventions, primary care health systems

## Abstract

**Introduction:**

The shift from in-person care to telemedicine made it challenging to provide guideline-recommended tobacco cessation care during the COVID-19 pandemic. We described quality improvement (QI) initiatives for tobacco cessation during the COVID-19 pandemic, focusing on African American/Black patients with high smoking rates.

**Methods:**

The QI initiatives took place in the San Francisco Health Network, a network of 13 safety-net clinics in San Francisco, California between February 2020 and February 2022. We conducted direct patient outreach by telephone and increased staff capacity to increase cessation care delivery. We examined trends in tobacco screening, provider counseling, and best practice for cessation care (ie, the proportion of patients receiving at least 1 smoking cessation service during a clinical encounter).

**Results:**

In-person visits at the onset of the pandemic was 20% in April 2020 and increased to 67% by February 2022. During this time, tobacco screening increased from 29% to 74%. From March 2020 to March 2021, 34% more patients received provider counseling by telephone than in-person. The trend reversed from April 2021 to February 2022, where 23% more patients received counseling in-person than by telehealth. Best practice care increased by 23% from June 2020 to February 2022: 24% for African American/Black patients and 23% for other patients.

**Conclusions:**

Telehealth adaptations to the EHR, targeted outreach to patients, and a multi-disciplinary medical team may be associated with increases in cessation care delivery during the COVID-19 pandemic.

## Introduction

In 2019, an estimated 34.1 million adults (14%) reported tobacco use in the United States (US).^1,2^ Tobacco use is concentrated in low-income populations, including some racial/ethnic minority groups who report higher rates of tobacco use than the general population.^1^ Despite smoking fewer cigarettes per day, African American/Black individuals who smoke experience higher morbidity and mortality from tobacco use than White individuals.^3^

Populations with high medical comorbidities and social needs seek healthcare in safety-net health systems, where the economic toll of smoking is high.^4,5^ Medicare and Medicaid contribute nearly 60% ($109 billion) of the annual health care costs from smoking-related conditions.^6^ A 1% reduction of smoking prevalence is associated with $2.5 billion in annual Medicaid savings,^7^ highlighting a role for safety-net health systems to increase delivery of guideline-recommended cessation programs.^8^

Safety-net health systems that are community-based and patient-centered are best poised to serve patients with high social and medical needs.^5,9,10^ These health systems deliver comprehensive care through medical teams that include healthcare providers, pharmacists, psychologists, social workers, and ancillary staff.^11,12^ Medical teams can use their electronic health records (EHR) to receive automatic reminders for tobacco screening,^13^ refer for smoking cessation services (eg, behavioral counseling and pharmacotherapy),^14^ and monitor receipt of services.^15,16^ While health systems have optimized their in-person delivery of smoking cessation care, they have had to make adaptations for telehealth during the COVID-19 pandemic.^17-20^

Studies have highlighted a role for telehealth in providing cessation care,^21^ particularly during the pandemic when health systems resources were constrained. In Virginia, a military health facility used social media, email, and online patient portals to inform patients of cessation resources.^22^ After transitioning to a telephone tobacco counseling program, a cancer center in New York reported improved patient engagement via telephone more so than in-person counseling.^23^ However, less is known about how safety-net systems have delivered cessation services while adapting to telehealth during the COVID-19 pandemic.

In this study, we describe telehealth adaptations and quality improvement (QI) initiatives to improve delivery of tobacco cessation services in a safety net health system in San Francisco, CA, during the COVID-19 pandemic. We conducted direct patient outreach by telephone and improved staff capacity to deliver cessation care. We examined trends in tobacco screening, provider counseling, and best practice for delivery of smoking cessation care. Given the high rates of tobacco use among Black/African American patients in our safety net health systems, we examined differences in delivering best practice care for Black/African American versus other patients.

## Methods

### Setting and Participants

The San Francisco Health Network (SFHN) annually serves nearly 60 000 low-income and racially/ethnically diverse patients, including over 7000 patients who smoke, across 13 primary care clinics. Of the 13 clinics, 4 were academic practices housed in a university-affiliated public hospital (smoking prevalence 7%-34%). The other 9 were community health clinics located across San Francisco (smoking prevalence 10%-41%). We extracted data on tobacco screening and delivery of cessation services from the EHR between February 2020 and February 2022. The University of California, San Francisco Institutional Review Board (#18-26398) considered the study exempt.

### Tobacco Cessation Care Model Before the COVID-19 Pandemic

Before the pandemic, medical teams delivered smoking cessation services in person. Medical assistants (frontline staff) screened all patients for tobacco use and referred those who smoked to Kick It California, formerly known as the California Smokers’ Helpline (“Helpline”).^24^ Healthcare providers counseled patients and prescribed cessation medications during clinical encounters. Behavioral assistants (ancillary staff at the health coach level) provided cessation coaching using motivational interviewing to patients who were referred to them by medical assistants and healthcare providers. Members of the medical team were located in the same clinic, allowing for easy coordination of cessation care through in-person hand-offs between team members. We also built a single-click option within the EHR for each clinical staff to document tobacco screening and delivery of cessation services during each clinical encounter.

In August 2018, we built a general tobacco registry embedded within the EHR that included a list of all smoking patients who had not received cessation care in the past 2 years, a pay-for-performance metric for tobacco.^25^ The registry included demographic information, current smoking status, the most recent primary care visit date, and the dates when they received cessation services (eg, medical assistant referral, healthcare provider, and behavioral assistant coaching). The medical team accessed the tobacco registry to reach patients during and between clinic visits. We operationalized the registry by (1) populating the registry data, (2) validating the registry data by chart review, (3) creating lists of patients who had not received services, and (4) using the lists to make practice changes (eg, training medical teams to document delivery of counseling in the EHR).

### Tobacco Cessation Care Model During the Pandemic

During the pandemic, clinic operations transitioned to a telehealth model, with staff and providers delivering care by telephone. To adapt to this model, we adopted new practices to ensure that cessation services were provided and documented appropriately in the EHR. We conducted direct patient outreach by telephone and improved staff capacity to deliver cessation care via telephone encounters.

#### Patient outreach

**Figure 1.**
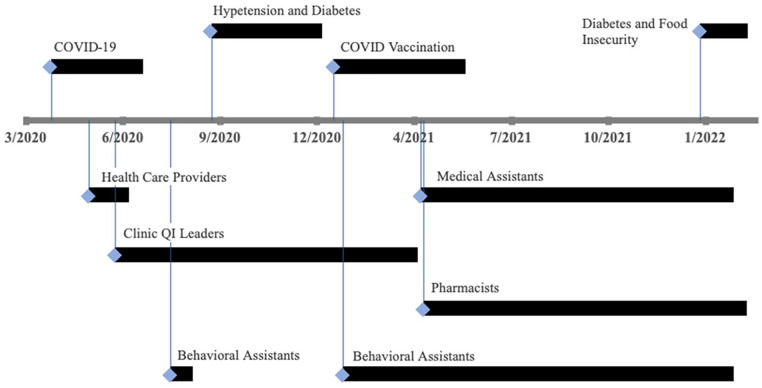
Timeline of quality improvement initiatives to increase delivery of tobacco cessation services at the San Francisco Health Network during the COVID-19 pandemic. Patient outreach efforts are above the timeline axis, staff capacity building efforts are below the axis.

We developed a patient registry that included all adult patients at risk of contracting COVID-19 due to medical comorbidities (ie, congestive heart failure, chronic obstructive pulmonary disease, asthma, diabetes, or HIV). We included information on patient demographics and smoking status in this registry, recognizing that each outreach attempt to patients was an opportunity to also discuss tobacco use. Medical assistants conducted telephone outreach to patients in the registry to inform them about COVID-19 services, and referred those with medical or behavioral health needs to an appropriate clinical team member. Patients in the registry who were current smokers also received resources for smoking cessation including a referral to the Helpline during the outreach phone call. We used this same process for other patient outreach efforts during the COVID-19 pandemic. For example, we conducted a telephone outreach to patients who had uncontrolled diabetes and hypertension, who had food insecurity, and to increase COVID-19 vaccinations, and with each of these outreach efforts, patients who were smokers also received resources for smoking cessation (Figure 1).

#### Clinic staff capacity building

To increase staff capacity to deliver cessation care, we trained each medical team member and clinic QI leaders (Figure 1). Medical assistants received training on how to document patients’ smoking status in the HER, and how to provide a cessation referral. Healthcare providers and pharmacists received training on how to provide and document cessation counseling in the HER. Behavioral assistants received training on how to access a “missed opportunities” list in the HER, that is, a list of patients who did not receive a cessation service during their primary care visit for proactive telephone outreach. Behavioral assistants also received training on how to provide motivational interviewing for cessation coaching, and how to document the telephone outreach in the HER. Clinic QI leads like medical directors, nurse managers, and QI data analysts received training on how to access data on missed opportunities to provide cessation care, and staff performance around smoking cessation. We shared weekly data on cessation referral and counseling to each of the 13 clinics. The data allowed clinics to examine their progress over time, identify opportunities to make improvements, and assess their performance in comparison to other clinics in the network. These capacity building efforts were ongoing from March 2020 to February 2022. The SFHN uses the plan-do-study-act cycle^26^ as a QI framework to develop, test, and implement practice changes throughout the health system. We used the same QI framework to develop, test, and implement these outreach and capacity building initiatives during the COVID-19 pandemic.

### Measurements

We examined trends in 3 metrics between February 2020 and February 2022: (1) tobacco use screening, (2) healthcare provider counseling, and (3) best practice in delivering cessation care. We defined “best practice” as delivering at least 1 smoking cessation service (ie, medical assistant referral, healthcare provider counseling, behavioral assistant coaching, and pharmacotherapy) during a primary care visit. We set the goal for best practice at 60% (ie, each week, at least 60% of patients who smoked received a cessation service during their visit), which was the average for the top 7 performing clinics in June 2020. Because best practice is an aggregate measure of all cessation services, we used this metric to assess delivery of cessation services between African American/Black and other patients.

## Results

### Receipt of Cessation Services Before and After Adaptations for Telehealth

In March 2020, the tobacco registry included 6,884 adults who smoked, of whom 64% (N = 4425) had received at least 1 cessation service in the past 2 years. By the end of the study (February 2022), the registry included 9593 patients who smoked, of whom 76% (N = 7336) received at least 1 cessation service in the past 2 years.

### Primary Care Visits and Delivery of Tobacco Screening

**Figure 2.**
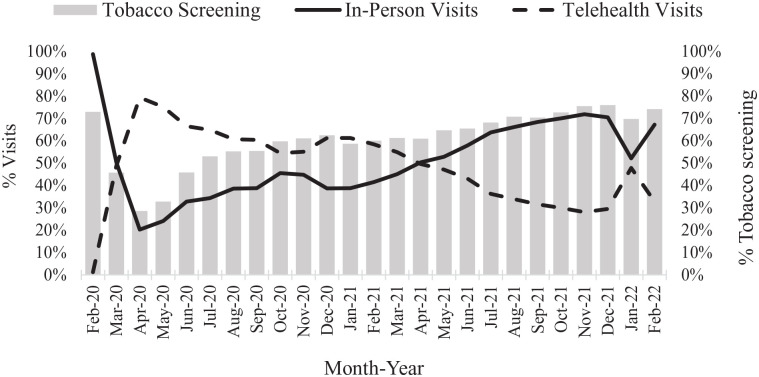
Trends in in-person and telehealth primary care visits and tobacco screening during primary care visits from February 2020 to February 2022.

In February 2020, there were a total of 10,042 primary care visits, of which 99% were in person (Figure 2). The percentage of in-person visits dropped at the onset of the pandemic and was at its lowest (20%) in April 2020. By February 2022, the in-person visits gradually increased to 67%. Conversely, only 1% of primary care visits were conducted by telephone in February 2020. However, telehealth visits were at their highest (80%) in April 2020, and gradually decreased to 33% in February 2022. In February 2020, tobacco screening was 73%, and declined to 29% in April 2020 in parallel with a decline in in-person visits. Over the study duration, with adaptations to the telehealth model, tobacco screening increased to 74% by February 2022.

### Delivery of Healthcare Provider Counseling

**Figure 3.**
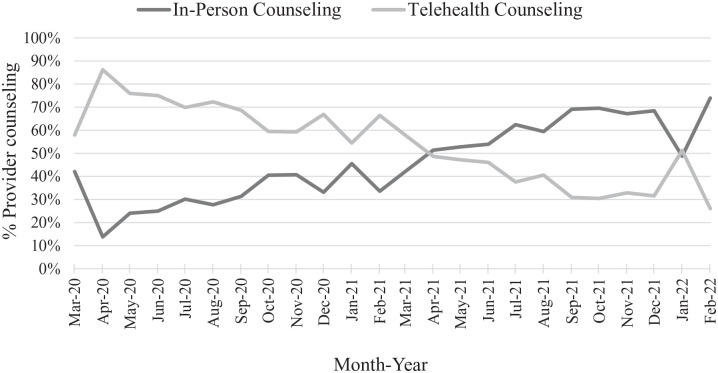
Percentage of in-person and telehealth counseling among those who received healthcare provider counseling during primary care visits from March 2020 to February 2022.

Telehealth counseling was the primary method of provider counseling between March 2020 to March 2021; on average, 34% more patients received provider counseling over the telephone than in-person (Figure 3). However, the trend reversed between April 2021 and February 2022 as more primary care visits were taking place in person. From April 2021 to February 2022, an average of 23% more patients received in-person counseling than telehealth counseling.

### Delivery of Best Practice Cessation Care

**Figure 4.**
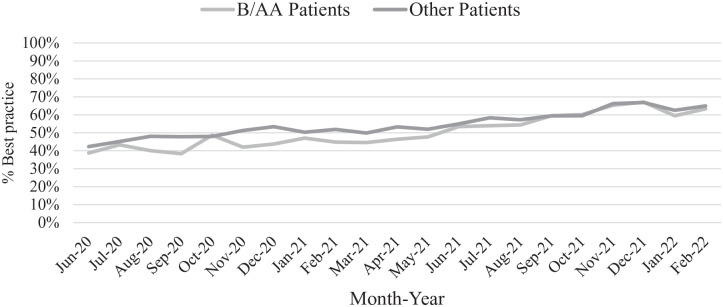
Trends in best practice for smoking cessation care for African/American Black and other patients from June 2020 to February 2022. Best practice for smoking cessation care includes providing at least 1 cessation service during a primary care encounter.

In June 2020, best practice care for all patients was 41%: 39% for African American/Black and 42% for other patients (Figure 4). By February 2022, best practice care increased to 64%: 63% for African American/Black patients and 65% for other patients. Best practice care increased by 23% between June 2020 and February 2022. Between September 2021 and February 2022, the gap in best practice care for African American/Black patients and other patients appeared to have closed.

## Discussion

From March 2020 to February 2022, the SFHN implemented telehealth adaptations and QI initiatives to improve delivery of tobacco cessation care during the COVID-19 pandemic. With patient outreach and staff capacity building efforts, we saw a 12% increase (64%-76%) in the percentage of tobacco registry patients who received at least 1 cessation service. Tobacco screening increased by 45% (29%-74%) and best practice increased by 23% with no observed disparity in the delivery of cessation care between African American/Black and other patients during the latter 6 months of the pandemic.

The SFHN has a multidisciplinary population health team that monitors the SFHN’s performance on several primary care measures, including tobacco screening and counseling.^19,27,28^ The SFHN also had a dedicated tobacco coordinator trained in clinic-facing tasks and data analytics. The coordinator worked with the information technology (IT) staff to build, operationalize, and improve the tobacco-focused disease registries. The coordinator’s clinic-facing tasks included building rapport with clinic staff and leadership to support the use of the disease registries, train staff on providing counseling and documenting efforts in the HER, and assist clinics in meeting the 60% best-practice goal. The population health team, including dedicated staff to address tobacco use, and the IT infrastructure were critical to transitioning cessation services in the SFHN to a telehealth model.

While there were some differences in delivery of cessation care between African American/Black and other patients during the pandemic, these gaps appeared to close toward the end of the study time frame. Clinics that served a higher proportion of African American/Black patients were located in neighborhoods that were heavily impacted by COVID-19. The SFHN deployed staff in those clinics to COVID-19 operations, and they had less capacity to focus on tobacco cessation services even as they saw a large volume of African American/Black patients who smoked. These factors may have contributed to differences in best practice care between African American/Black patients and other patients. As COVID-19 operations reduced, those clinics shifted resources to QI efforts, dedicating staff as QI leaders to address gaps in care. To further support these clinics, the tobacco coordinator and population health team provided consultations weekly and on an as needed basis on how to use clinic-level data to address missed opportunities in providing cessation care and to improve best practices. These efforts may have contributed to the overall increase in best practice and a decrease in the gap in cessation services for African American/Black and other patients.

The tobacco coordinator regularly engaged with medical assistants and their supervisors to share weekly staff performance data on the number of patients screened and counseled, and to reinforce training on documentation of tobacco screening and cessation referrals. We found that healthcare providers reported having less capacity to focus on tobacco cessation counseling during the COVID-19 pandemic. Our findings highlight a role for cessation efforts that do not solely rely on staff to integrate cessation counseling into primary care. Interventions that use the HER to submit electronic referrals to the Helpline hold promise for increasing access to cessation counseling for some patients in the safety-net health system.^29,30^

There are several limitations to our study. As the purpose of QI is different from that of clinical research, our QI interventions did not rely on randomized controlled trial methods or a control group. Instead, we continuously evaluated and modified interventions using PDSA cycles.^26^ Like tobacco, other primary care metrics such as cancer screenings declined at the onset of the pandemic.^31^ Although we cannot draw causal inferences between the QI efforts described in this study and tobacco cessation services, we observed an increase in tobacco screening and counseling that could be associated with these efforts. Simultaneously, other primary care metrics that the SFHN did not address systematically through QI efforts declined further or stayed the same. Smoking status was self-reported, potentially leading to a misclassification bias. However, the population health team validated the HER data by manual chart review to ensure that the quality of smoking assessments and receipt of counseling were accurate, reducing the potential for misclassification bias.^28^

In conclusion, we found that adaptations to the EHR and team-based care model can increase telehealth delivery of tobacco cessation services in a large safety-net health system. The adaptations relied on a multidisciplinary team to implement the changes and facilitate rapid QI cycles. Proactive telephone outreach may offer cessation referrals to some patients, but other methods are needed to increase delivery of cessation counseling within safety-net systems. Targeted and personalized interventions that dynamically adapt to the needs and context of each clinic may more effectively improve health system services and tobacco-related health equity.
